# Multi-national perceptions on challenges, opportunities, and support structures for Dual Career migrations in European student-athletes

**DOI:** 10.1371/journal.pone.0253333

**Published:** 2021-06-25

**Authors:** Philip X. Fuchs, Mojca Doupona, Kinga Varga, Marta Bon, Cristina Cortis, Andrea Fusco, Loriana Castellani, Niko Niemisalo, Heikki Hannola, Patrice Giron, Jörg Förster, Laura Capranica, Herbert Wagner

**Affiliations:** 1 Department of Sports and Exercise Science, University of Salzburg, Salzburg, Austria; 2 Department of Athletic Performance, National Taiwan Normal University, Taipei, Taiwan; 3 Faculty of Sport, University of Ljubljana, Ljubljana, Slovenia; 4 Department of Human Sciences, Society and Health, University of Cassino and Lazio Meridionale, Cassino, Italy; 5 Department of Responsibility in Business and Services, Lapland University of Applied Sciences, Rovaniemi, Finland; 6 University Sport Service Hamburg, University of Hamburg, Hamburg, Germany; 7 Department of Movement, Human and Health Sciences, University of Rome Foro Italico, Rome, Italy; 8 European Athlete as Student, Malta; University of Illinois at Urbana-Champaign, UNITED STATES

## Abstract

Despite Dual Careers (sports and education) and mobility of students being priorities in the funding policies of the European Commission, migrating student-athletes report severe challenges and decreased performance or dropouts at sport and academic levels. The objective of this study was to depict and assess the perceptions on challenges, support services, and their effectiveness in consideration of specific characteristics of participants and migrations. Based on a meta-synthesis and previous findings, a 50-items questionnaire was developed and completed by 245 student-athletes in 5 European countries. Participants with Dual Careers migration experience (*n* = 140) were considered for analyses of qualitative and quantitative (ordinal 5pt-Likert-scaled and metric) data on the Dual Career status, migration characteristics, received services, and outcomes. Chi-square-tests were conducted for differences between countries and genders at a significance level of *p* < .05. Country-related differences were found for experiences and intentions to migrate (*X*^2^_(12)_ = 50.52, *p*<0.001), duration of the migration (*X*^2^_(16)_ = 38.20, *p* = 0.001), financial support (*X*^2^_(8)_ = 29.87, *p<*0.001), and decreased performances in academics (*X*^2^_(16)_ = 56.12, *p<*0.001) and sports (*X*^2^_(16)_ = 31.79, *p* = 0.01). Gender-related difference emerged in financial support (*X*^2^_(4)_ = 10.68, *p* = 0.03), duration of the migration (*X*^2^_(4)_ = 14.56, *p* = 0.01), and decreased academic performance (*X*^2^_(4)_ = 10.57, *p* = 0.03). Tutoring and counselling support was ranked as the most effective support, especially when received from the academic field (4.0±1.0 pt) and others (4.1±0.8 pt), followed by online services from sport and academic sectors (both: 3.9±0.9 pt). Considering the pervasive globalization of sport and education, Dual Career migration can contribute to the development of a European sport culture. The high ratio of migrating student-athletes underlines the relevance of migrations in the field of Dual Careers. This study contributes to the literature by adding insights on practices, challenges, supports, and outcomes perceived by student-athletes migrating in Europe. Moreover, country- and gender-related differences support the consideration of specific characteristics and reveal critical factors in specific target groups. The findings contribute to identifying requirements and effective support measures in Dual Career migrations and can be used to improve support services.

## Introduction

Despite the increase of sports globalization that highly relies on the mobility of athletes, there is limited evidence on the actual challenges, needs, and possible solutions for European Dual Career (DC) of student-athletes with a long-term geographic (national and/or transnational) migration due to work, athletic, educational, or personal development and opportunities [1–3]. The existing situation persists even though the mobility of people and geographical unity is one of the founding principles of the European Union (EU) [4], strongly supported by the Bologna process and the Member States contribution. Furthermore, due to the peculiarity of collegiate sport system in the U.S.A., transcontinental migration is an appealing option for European elite athletes [5]. In fact, this possibility is reinforced due to the different sports and educational contexts in Europe, the various socio-cultural influences, and the diverse DC approaches of the EU Member States that restrain the identification of clear transnational exchange opportunities for migrating student-athletes. In the framework of this investigation, Dual Career refers to the simultaneous pursue of a sport and an academic career. A student-athlete is understood as an actively competing athlete who is actively committed to an academic study program at tertiary education level.

Migration could be conceptualized as a time-space phenomenon, encompassing a permanent or semi-permanent change of residence within or to another country [6]. In this investigation, we define migration as a change of residence to another country independent on the duration of the stay. In general, interviews and surveys could be valuable means to investigate push factors operating within the home country initiating the student-athlete’s decision to relocate and pull factors operating within the host institution to make migration relatively more attractive than other potential destinations. In a globalized world, sports and education migration could allow to study migration as a form of social mobility in modern society, involving aspects related to institutions, organizations, networks, and social interactions [7].

Since 1987, the European Community Action Scheme for the Mobility of University Students (ERASMUS) program has financed the mobility of students within the EU to improve their European identity, European citizenship, and international competence. Furthermore, policy recommendations on educational mobility of European students have been provided based on empirical evidence due to distance, time, financial, educational, and cultural determinants affecting mobility [8]. Main findings included economic support, more interinstitutional agreements across borders, providence of language learning services, and better marketing of academic quality as a pull factor [8]. Despite the extended sports networks developed to search for talented athletes on a global scale [9], there is a limited research on international student-athlete mobility. Recently, transnational career development and transitions of six athletes in the Nordic countries have been investigated by means of an interview study [5]. Main finding was the identification of three patterns of transnational DC migration (i.e. sport exile pathway within the EU, sport mercenary pathway to the U.S.A., and nomadic cosmopolitan pathway to the U.S.A.). Due to the limited sample size and the geographical context of this study, the identified patterns are not exhaustive [5], and the findings are not comprehensive. There is a need to expand the knowledge, especially regarding cross-national and gender-related differences in educational, sports, and cultural environments and effective DC services for migrating student-athletes. This deficit is to be tackled by the current investigation.

Gender-related differences are important for further consideration because previous research has shown gender-dependent effects on DC [6,10]. For example, gender was reported to play a role for priorities and outcomes during DC [10] as well as for motivation to migrate and decision making to stay or return to the home country [6]. Greater commitment to academics [10], ability to manage academic requirements [10], and reluctance to return to the home country because of the fear of losing new-won freedom were reported in females. Interestingly, females were also reported to be over-represented in academic migration programs (i.e. ERASMUS) compared with males in almost all European countries [11]. Motivation is known to affect the prioritization of academic or athletic commitments and consequently the outcomes in the respective fields [12,13]. It is therefore recommended to account for it in the current investigation. Considering the heterogeneous DC policies [14], and home and host country characteristics of the Member States could determine different approaches to DC migration.

Different DC policies at national levels in the EU were documented [14–17] and were characterized as four-fold typology including state centric regulation, the state as a facilitator, national institutions negotiating on behalf of individuals, and ‘laisser faire’ (i.e. no support structures) [14]. These variations were expressed in differences in economic support, organizational regulations (e.g. at academic levels), and entry requirements (e.g. competitive level) [14]. To characterize different DC cultures, approaches, and praxis in Europe [14–17], EU partners from Austria, Finland, Germany, Italy, and Slovenia considered the perspectives of migrating student-athletes valuable to raise awareness and to generate novel research questions on migrating student-athletes. In this endeavor, an online survey on current opportunities for national and transnational exchange of student-athletes in the partner countries has been developed. Findings were expected to be valuable to inform national policy makers, DC stakeholders, and scholars for further implementation of DC policies and services specifically targeting student-athlete migration, considered central for the advancement of a DC culture in a globalized society.

Therefore, the objective was to fill the gap in the current literature (i.e. deficits in the migrating student-athletes’ experience and perception on challenges, practices, supports, and outcomes) and to assess the point of view of student-athletes from five European Member States with different DC policies in place. In particular, it was deemed relevant to display and describe 1) socio-demographic characteristics of migrating student-athletes to frame this population; 2) student-athletes’ motivation for migration and their support from academic institutions, sport bodies, and DC service providers; 3) student-athletes’ awareness of DC available support and their suggestions for DC improvements; and 4) the effects of country and gender.

It has been hypothesized that differences in migration experiences of student-athletes will emerge with respect to gender and country.

## Methods

This study was carried out in accordance with the Declaration of Helsinki upon approval of the Institutional Review Board of the Department of Human Sciences, Society and Health of the University of Cassino and Lazio Meridionale (approval number 14357.2019.06.18 date: May 8^th^ 2019). No minors were included in the sample and, therefore, no parental consent was required.

### Instrument

In line with the literature [18–20], the conceptualization of the factors that could contribute to facilitate or hamper DC migration of student-athletes was achieved. This included specifically the consideration of specific demographic characteristics, sport and university engagement, awareness, and support at various levels [18] as well as typology determined by push/pull factors (motivation) [20]. A meta-synthesis of 9 qualitative studies on DC was consulted to ensure that relevant aspects were covered by the instrument, based on third-order constructs (e.g. physical, psychological, and other conditions, barriers, social and financial aspects, and support) [19]. Applying the Focus Group method [21], a 50-item semi-structured questionnaire (S1 Table) was constructed in English. The Focus Group was implemented as a group interview where experts (i.e. long-term representatives of the European Athletes as Student network and national leading DC associations) exchanged and discussed on DC migrations, especially with respect of the aforementioned topics [18–20] and the goal to develop an instrument. The discussion was protocolled, summarized, and items were derived to reflect the themes. A similar approach was previously applied to develop a questionnaire in DC [10]. The following major themes of DC in migrating student-athletes were derived:

- Demographic characteristics of the sample (Q1-7), including the practiced sport, country, gender, age, university level, athletic level, and sport commitment;
- Migration experience (Q8);
- Characteristics and effectiveness of the financial support (Q9-18);
- Reasons for migration (Q19-23);
- Availability of tutoring, including information on providers (Q24-32);
- Availability of organizational and online support (Q33-36);
- Migration challenges and effects on performances (Q37-47);
- Awareness of good practices and possible suggestion for DC implementation (Q48-50).

The instrument included open, closed, and dichotomous (yes or no) responses. To measure the effectiveness of various services, a 5-point Likert-scale ranging from 1 (lowest level of agreement, e.g. ‘not at all’) to 5 (highest level of agreement, e.g. ‘absolutely’), with the lowest values (1 and 2) and the highest values (4 and 5) subsequently merged into distinct categories (i.e. disagreement, agreement). A 3-point response represented a neutral position. Furthermore, respondents were also allowed to elaborate further on their answers for questions related to envisage good practices and possible solutions for DC challenges related to migration. The single statements of these open responses were first paraphrased and then clustered as topics. The number of mentions informed about the importance of certain topics for the migrating student-athletes and supported the interpretation of closed responses.

Since language has an impact on responses and might obscure important differences between countries [22], the present cross-national study design deemed crucial to translate the questionnaire in Finnish, German, Italian, and Slovenian language. To produce a conceptual and semantic equivalent of the instrument, forward and backward translations were performed [23], and pilot interviews with students-athletes who experienced sport-related migration were carried out for validation and to verify the clarity of the instructions, items, and responses options. In two countries, minor translation-related modifications were implemented by the leading researchers in accordance with the pilot participants’ feedback until all potential misinterpretations were solved. The questionnaire was then considered suitable to be administered to student-athletes in Austria, Finland, Germany, Italy, and Slovenia. To reach migrating student-athletes of five the Member States (i.e., Austria, Finland, Germany, Italy, and Slovenia), a web tool was selected to allow multimedia and self-administration [24].

### Recruitment

The inclusion criteria for recruitment of participants encompassed: 1) to be enrolled in a higher education study program; and 2) to compete in sports organized in federation structures. To recruit respondents in compliance with the country-specific regulations and standards of general data protection regulations and safeguarding privacy rights of personal data, a pre-notification email providing information on the online survey for migrating student-athletes and its link was prepared and sent to local/regional/national references (academic staff, sport staff, and DC service providers) who were required to recruit the target population of student-athletes [25,26].

During a 3-months period, each partner administered the questionnaire to student-athletes in the country. There were three rationales for the inclusion of student-athletes who did not migrate yet: 1) The authors wanted to avoid erroneous exclusion due to the term ‘migration’, which could be misinterpreted by the participant or collaboration partners (e.g. clubs, federations) who were involved in the recruitment process, 2) to assess the relevance of migration for the participants, the authors aimed to obtain the ratio of student-athletes who have already migrated or are willing to, and 3) the authors wanted to establish contact with all student-athletes for future support programs. However, data on experiences and practices were only analyzed for migrating student-athletes. The online survey was set-up in a way that student-athletes received a shortened questionnaire when they had no migration experience.

The partners adopted different strategies to reach student-athletes, dependent on the existence of national DC agencies and data bases:

- The University of Salzburg included KADA in distributing the online link of the questionnaire via e-mail to all KADA athletes in the national data base (713 in 2017). Given the limited number of responses after two months (despite two reminders to all KADA athletes), academic staff at other institutions, KADA athletes and student-athletes without KADA registration were contacted personally to fill the questionnaire. Total questionnaires collected: 45.
- Lapland University of Applied Sciences implemented the distribution of the questionnaire in cooperation with the Finnish Olympic Committee, regional sport academies, and teaching staff. Total questionnaires collected: 48.
- University Sport Service Hamburg distributed the questionnaire link: i) directly to athletes who participated on behalf of the University Sport Service Hamburg in international events in 2018 and to athletes enrolled in the program ‘Spitzensport Stipendium Metropolregion Rhein Neckar’, ii) to the New German athletes union, ‘Athleten Deutschland’, asking to promote the survey, iii) to the Olympic Training centers Hamburg/Berlin that refused to distribute the questionnaire. Total questionnaires collected: 46.
- University of Ljubljana distributed the online-link of the questionnaire via: i) the official list of student-athletes registered with SUSA (Slovenian University Sport Association) that includes students competing in University games, ii) the website of University of Ljubljana (Faculty of Sport), iii) individual students-athletes attending academic lectures, asking to administer also to other student-athletes. Total questionnaires collected: 42.
- Lacking a national database of student-athletes, University of Cassino and Lazio Meridionale prepared an explanation letter asking to promote and distribute the questionnaire via e-mail along with the online link. The letter was sent to: i) institutions that have established DC programs, ii) colleagues at other institutions known personally, iii) national sport federations, iv) coaches known personally, and v) student-athletes known personally. The questionnaire link was also shared via Instagram and Facebook. Total questionnaires collected: 64.

Participation was voluntary and anonymous. At the beginning of the questionnaire, the athletes were informed that they could withdraw from the study at any time without providing any reason. Informed consent was obtained when participants accepted the conditions and filled the online survey. Over a period of three months, an online data collection via quota sampling by means of a non-probability sampling method was achieved. However, these procedures did not allow calculation of the probability and response rates, being a not-list-based survey [24].

### Statistical analysis

Data were analyzed using the Statistical Package for the Social Science, version 24.0 (SPSS Inc., Chicago Illinois). To display socio-demographics, motivation, support, and awareness in student-athletes, descriptive statistics encompassed means and standard deviations and frequency of occurrence expressed in percentages for which a single response or multiple responses were allowed. For inferential statistics, the Chi-Square test (*X*^2^) was applied to ascertain differences between genders and countries. In cases of violation of Chi-Square assumptions, likelihood ratio results were provided. In the case of significant group effects on nonparametric data between countries, Mann-Whitney-U-tests were performed for single countries and reported if significant. Significance level was set at *p* < .05. Due to the limited number of given open responses, recommendations were not quantified but were qualitatively considered during the interpretation of the results.

## Results

### Demographic characteristics of the sample (Q1-7)

The sample was evenly distributed between countries (range: 17–26%). Details on gender, age, and studies were presented in Table 1. The participating student-athletes competed in ball sports (43%), athletics (21%), water sports (18%), combat sports (11%), skiing (11%), shooting and archery (6%), and aesthetic sports (2%), committing 17.4±9.5 hours per week to sports. No difference in the academic level emerged between genders (*X*^2^_(2)_ = 1.71, *p* = 0.43) but between countries (*X*^2^_(8)_ = 19.81, *p* = 0.01).

**Table 1:** Demographic characteristics of the sample.

	Italy (*n* = 64)	Finland (*n* = 48)	Austria (*n* = 45)	Germany (*n* = 46)	Slovenia (*n* = 42)	TOTAL (*n* = 245)
**Gender**						
Females	52%	40%	60%	83%	57%	58%
Males	48%	60%	40%	17%	43%	42%
**Age** [years]	23.2±3.2	24.0±3.0	22.7±2.8	22.8±4.5	24.0±5.8	23.4±4.0
**Studies**						
Bachelor	75%	67%	64%	75%	64%	69%
Master	25%	25%	36%	15%	24%	25%
PhD	-	8%	-	10%	12%	6%

### Characteristics of the migrating student-athletes (Q8)

**Fig 1:**
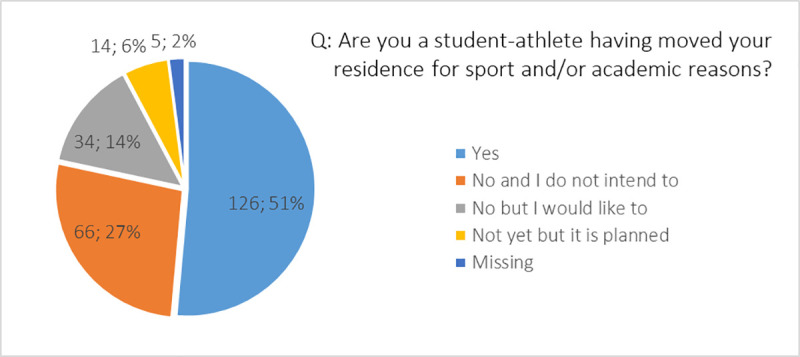
Distribution of respondents (*n*; %) with respect to migration experience and intentions.

With respect to the proportion of athletes that did not and do not intend to migrate (27%), the majority of athletes already experienced migration (51%), are going to migrate (6%) or would like to migrate (14%) in a future (Fig 1).

**Fig 2:**
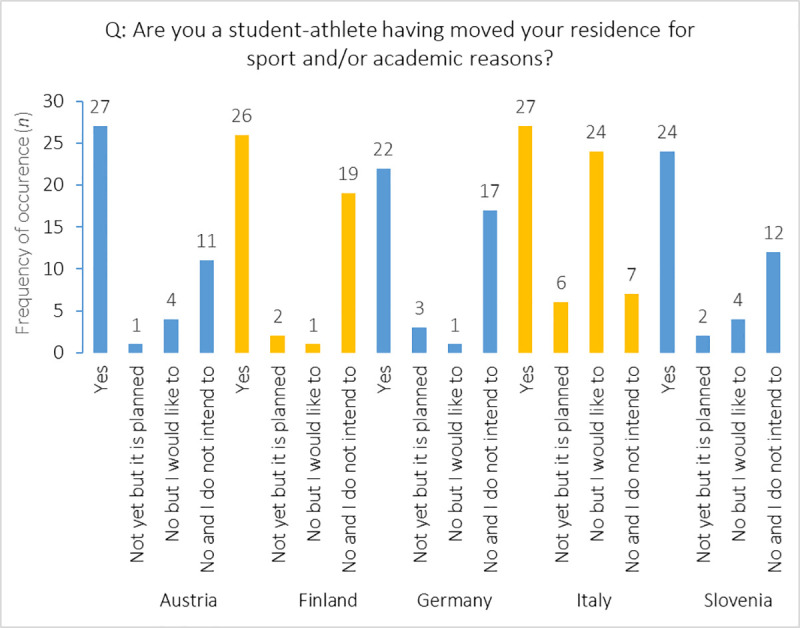
Country-related distribution of student-athletes (*n*) with respect to migration experiences and intentions.

Whilst no gender-related differences were found (*X*^2^_(3)_ = 0.29, *p* = 0.96), country-related differences (*X*^2^_(12)_ = 50.52, *p*<0.001) emerged for the migrating sub-samples (Fig 2). Compared with Austrian, Finnish, German, and Slovenian counterparts, Italian athletes showed the largest number of those who were willing to migrate in the future. Furthermore, 40% of Finnish and German student-athletes did not intend to migrate, whereas it was 29%, 26%, and 11% in Austria, Slovenia, and Italy, respectively.

**Fig 3:**
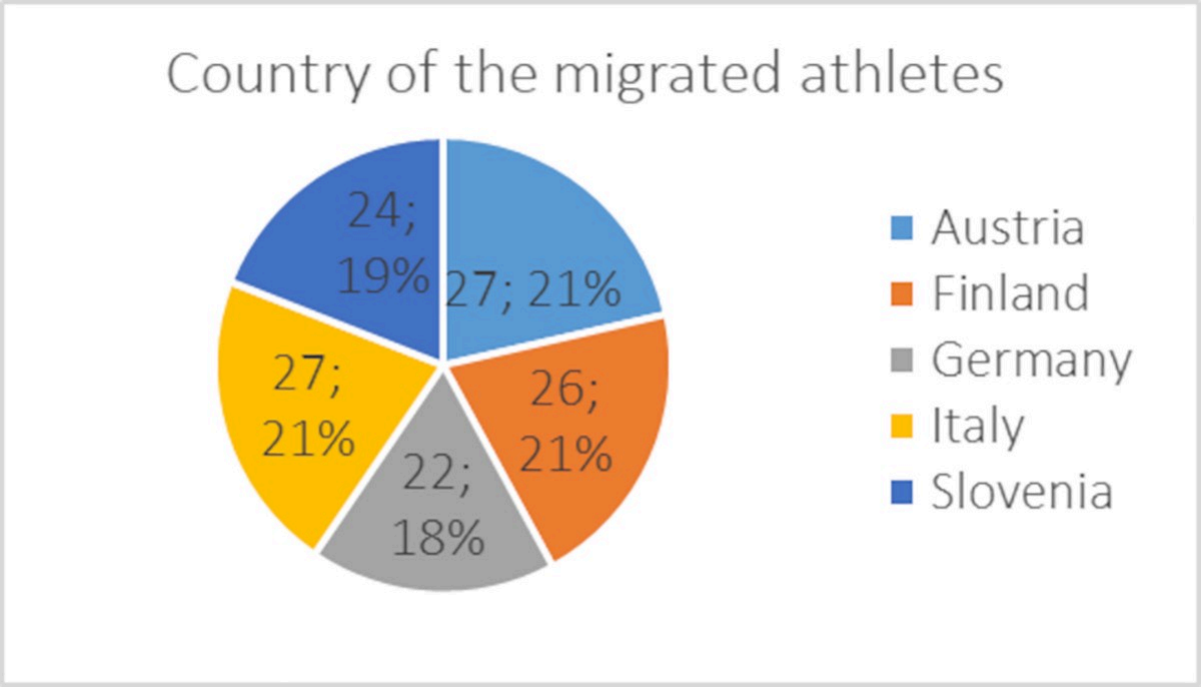
Country-related distribution (*n*; %) of migrating student-athletes.

With respect to the distribution of the whole sample, the proportion of athletes who experienced migration showed similar country-related (Fig 3; percentages refer to responses within the single countries) and gender-related (females: 41%; males: 59%) distributions. The 21–23 years (37%) and 24–26 years (36%) were the most represented age groups, followed by the 18–20 years (21%) and 27–29 years (6%) counterparts.

### Characteristics and effectiveness of financial support (Q9-18)

**Fig 4:**
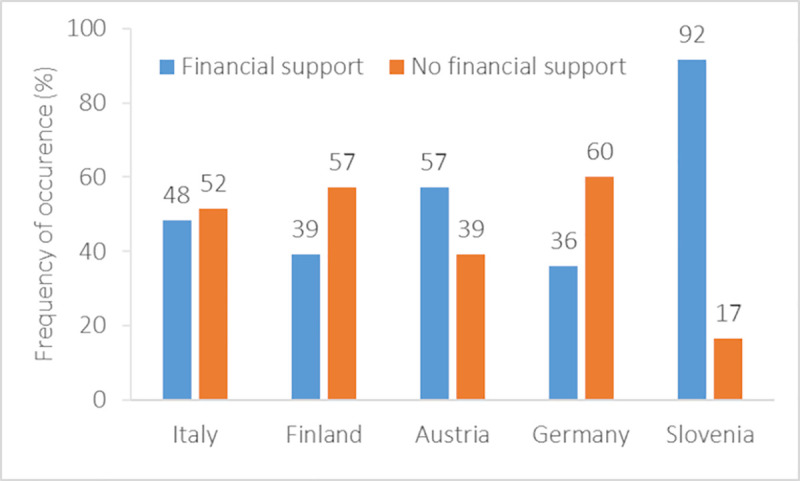
Frequency of occurrence (%) of financial support in relation to the country of the student-athletes.

Financial support was available only for 55% of the migrating student-athletes. A country-related difference was found (Fig 4) with higher occurrence of financial support for Slovenian (92%) student-athletes than for Germans (*U* = 165.00, *p*<0.01), Finnish (*U* = 197.00, *p*<0.01), Austrians (*U* = 262.00, *p*<0.05), and Italians (*U* = 274.00, *p*<0.01) (range: 36–57%) (*X*^2^_(8)_ = 29.87, *p<*0.001). Males (26%) received more frequently financial support than females (16%) (*X*^2^_(4)_ = 10.68, *p* = 0.03). Among the 74 migrated student-athletes who received financial support, the sources were stated to be academic institutions (28%), sports bodies (66%), DC systems (14%), and others (68%). Others included family (45%), sponsors (16%), national funds (9%), sports clubs (9%), and combinations of family, club, sponsors, and national funds (20%).

**Fig 5:**
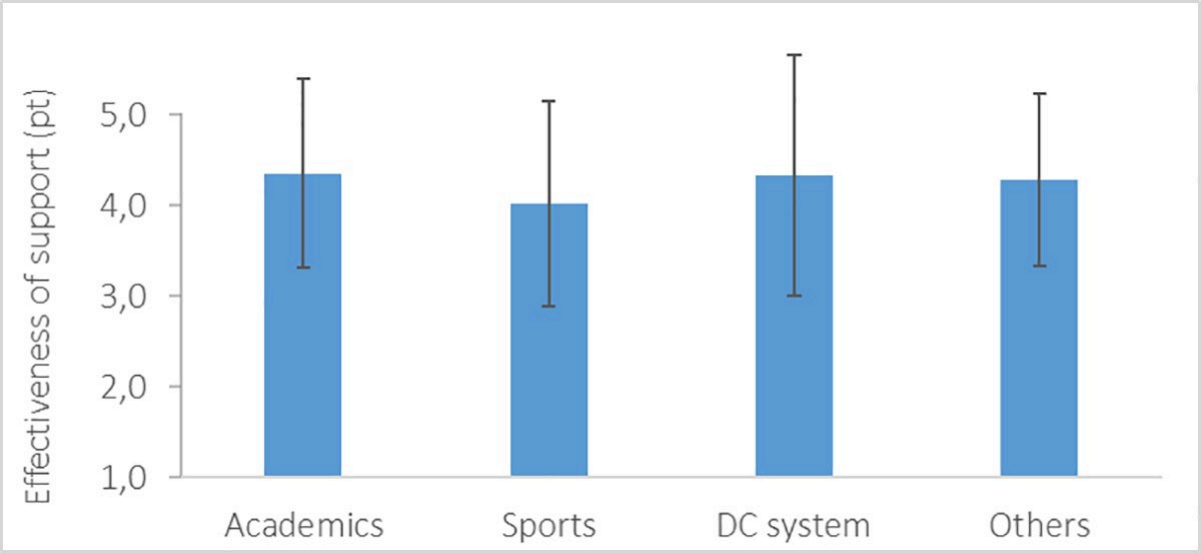
Mean and standard deviation of effectiveness of the financial support received by the migrating student-athletes.

In general, the respondents considered the received financial support very helpful (Fig 5), with effectiveness scoring between 4.1 and 4.4 pt in average for various sources of support.

### Reasons for migration (Q19-23)

**Fig 6:**
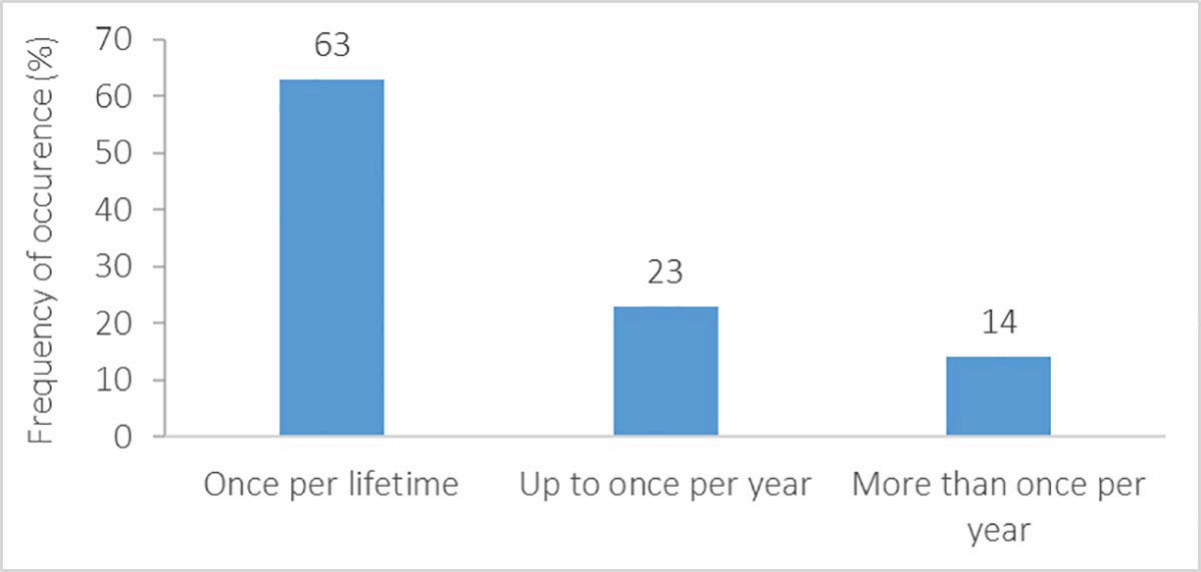
Frequency of relocation of migrating student-athletes.

**Fig 7:**
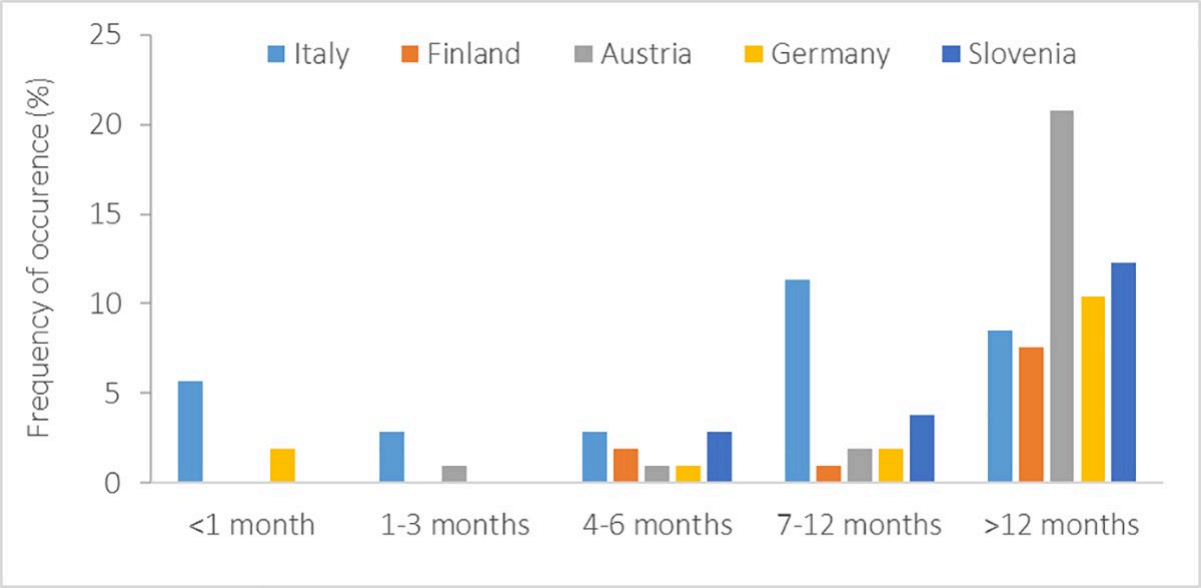
Duration of relocation of migrating student-athletes.

**Fig 8:**
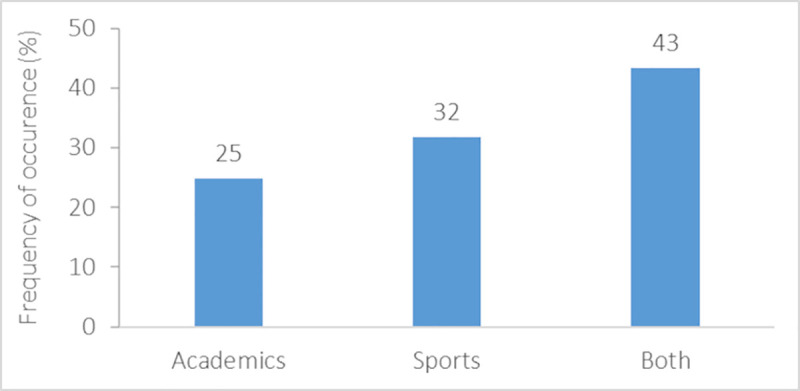
Reasons for relocation of migrating student-athletes.

Most migrating student-athletes experienced relocation only once in their life (63%). Almost a quarter (23%) frequently relocated up to once per year, and 14% more than once per year (Fig 6). Whilst a large portion of respondents (62%) relocated for longer than 12 months, a country-related difference (*X*^2^_(16)_ = 38.20, *p* = 0.001) with only Italian student-athletes relocating more frequently for 7–12 months compared with Austrians (*U* = 177.00, *p*<0.001), Finnish (*U* = 96.00, *p*<0.05), Germans (*U* = 162.00, *p*<0.01), and Slovenians (*U* = 186.00, *p*<0.01) (Fig 7). Females reported to migrate for shorter periods than males (*X*^2^_(4)_ = 14.56, *p* = 0.01). Most respondents (43%) declared that they relocated for both educational and sports reasons (Fig 8), whereas 32% and 25% of the student-athletes relocated for sports or academics, respectively.

### Availability of tutoring (Q24-32)

**Fig 9:**
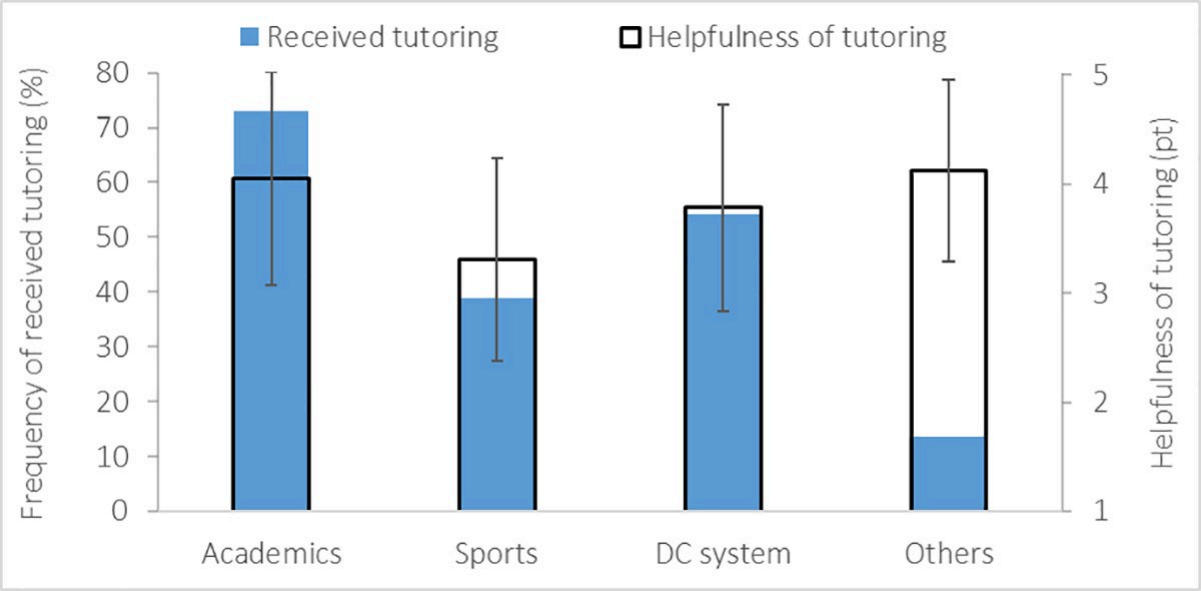
Mean and standard deviation of tutoring/counselling provided to student-athletes by academics, sports, Dual Career (DC) systems, and others (left vertical axis) and helpfulness of tutoring services if received (right vertical axis).

Only 47% of the migrating student-athletes received tutoring/counselling support. All of them received helpful tutoring from academics and others, also in combination with DC and sports services (Fig 9). In detail, student-athletes rated tutoring/counselling services to be more helpful when received from academics (4.0±1.0 pt) and others (4.1±0.8 pt) than from sports (3.3±0.9 pt) (Fig 9).

### Availability of organizational and online support (Q33-38)

**Fig 10:**
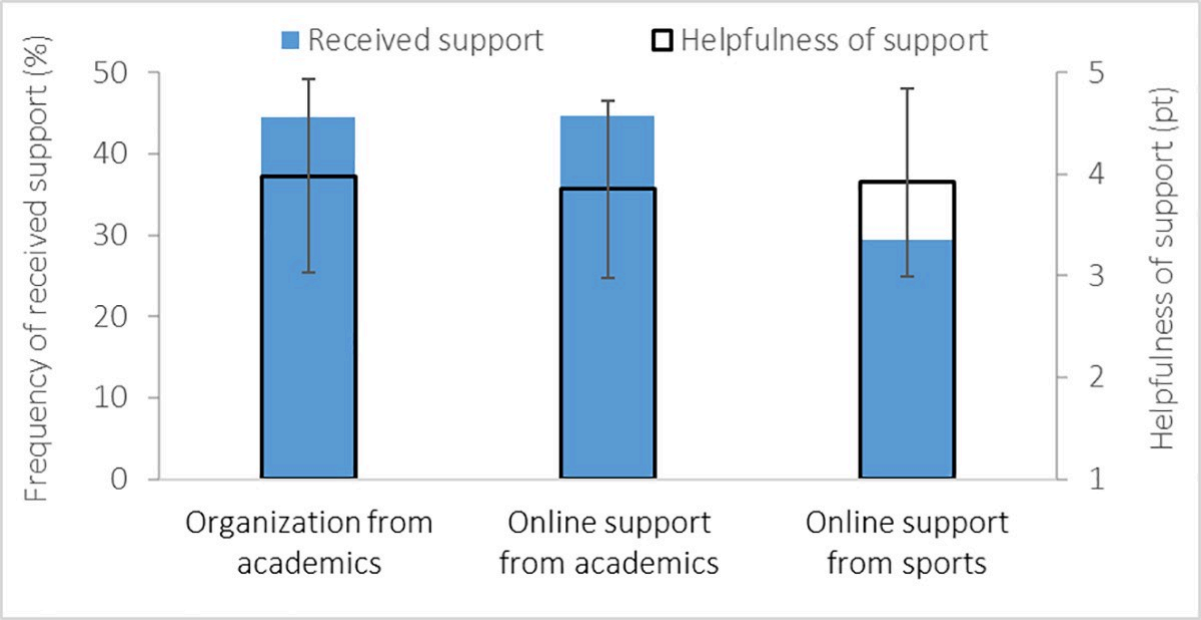
Mean and standard deviation of organizational and online support provided by academics and sports (left vertical axis) and helpfulness of organizational and online support if received (right vertical axis).

Almost half (45%) of the migrating student-athletes received organizational support and online support from academics, and only 29% received online support from sports (Fig 10). In general, the student-athletes received a combination of support, which was considered helpful (academics organizational: 4.0±1.0 pt; academics online: 3.9±0.9 pt; sports online: 3.9±0.9 pt).

### Moving challenges and their effects on performances (Q39-47)

**Fig 11:**
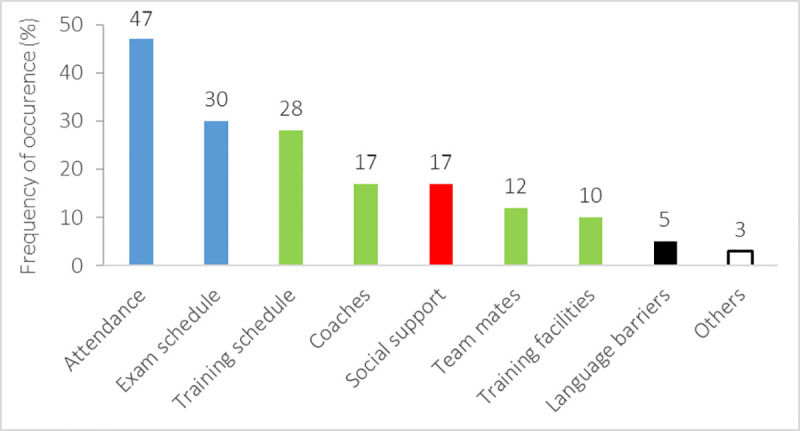
Frequency of occurrence (%) of the challenges reported by student-athletes because of migration, categorized as academic (blue), sports (green), social (red), language (black), and other (white) aspects.

Except for one respondent, all migrated student-athletes reported challenges because of migration (Fig 11), most frequently emerging in the academic section (class attendance: 47%; exam schedule: 30%) and the sports area (training schedule: 28%; coaches: 17%; teammates: 12%; training facilities: 10%) and social area (17%), followed by language barriers (5%). Furthermore, other challenges (3%) were reported, specifically pertaining financial problems, homesickness, and difficulty to study after training due to fatigue and capability to individual learning without attending the classes. Overall, the reported severity of challenges was limited (academics: 2.7±1.2 pt; sports: 2.5±1.3 pt; other areas: 2.4±1.1 pt).

Only 20% of the respondents (*n* = 18) maintained their performance at academics, sports, and other areas throughout the migration, whereas reported decreases in academics (3.2±1.0 pt) and sports (3.2±1.1 pt) performances were 70% and 63%, respectively. Furthermore, low social life (*n* = 2), self-esteem (*n* = 1), finance and health (*n* = 1), and time management were highlighted.

**Fig 12:**
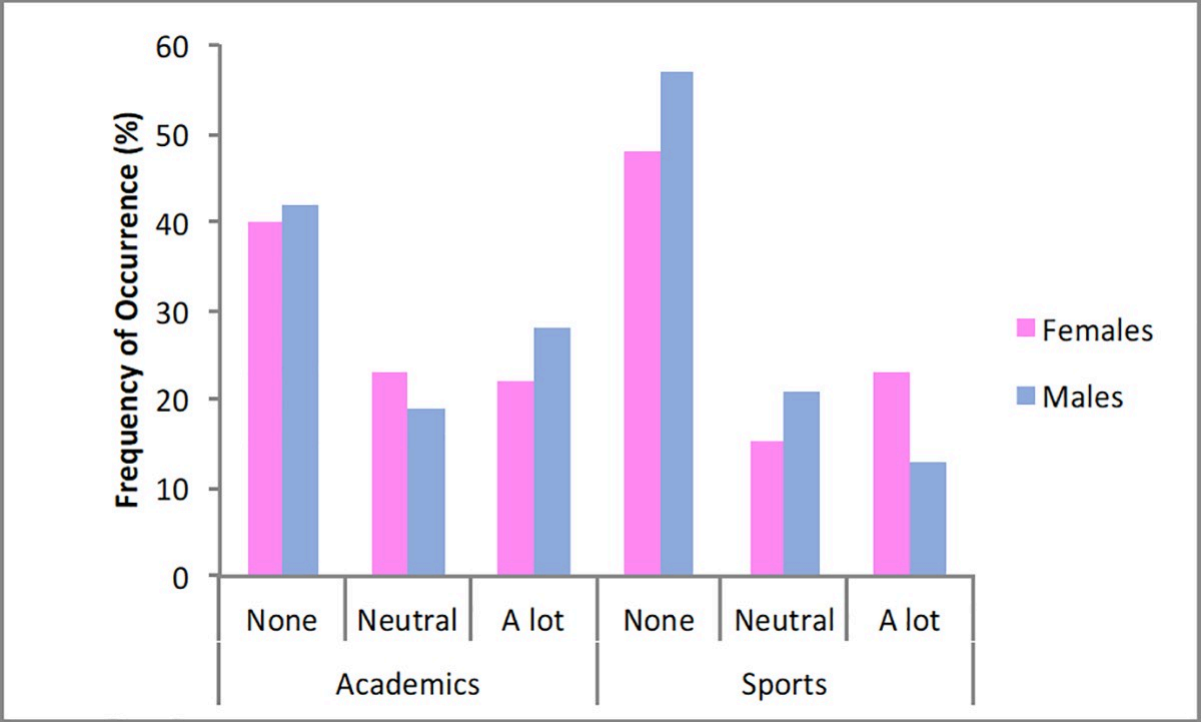
Frequency of occurrence (%) of decreased performances in relation to gender.

With respect to gender (Fig 12), females reported less serious decreases in academic performance (2.56 pt) compared with males (2.82 pt) (*X*^2^_(4)_ = 10.57, *p* = 0.03), whereas no effect was found for sports performance (females: 2.51 pt; males: 2.18 pt; *X*^2^_(4)_ = 2.83, *p* = 0.59).

**Fig 13:**
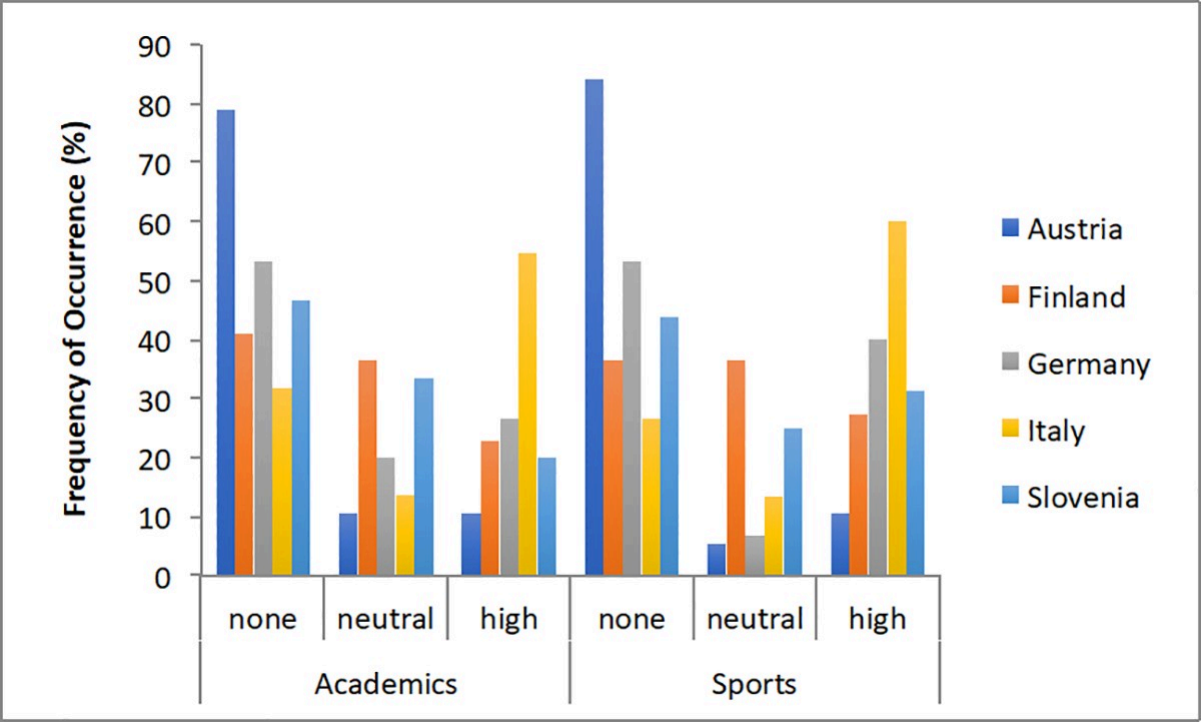
Frequency of occurrence (%) of decreased performances in relation to the country of the student-athletes.

In relation to the nationality of the athletes (Fig 13), differences emerged for the decreased performances in academics (*X*^2^_(16)_ = 56.12, *p<*0.001) and sports (*X*^2^_(16)_ = 31.79, *p* = 0.01). For academic performance decrease, differences were significant between Italy and Finland (*U* = 236.50, *p*<0.01), Italy and Austria (*U* = 112.00, *p*<0.001), Italy and Germany (*U* = 145.00, *p*<0.05), Italy and Slovenia (*U* = 127.00, *p*<0.05), Finland and Austria (*U* = 204.00, *p*<0.05), and Austria and Slovenia (*U* = 99.00, *p*<0.01). For sport performance decrease, differences were significant between Italy and Slovenia (*U* = 168.00, *p*<0.05), Finland and Austria (*U* = 200.00, *p*<0.05), and Austria and Slovenia (*U* = 108.00, *p*<0.01) Whilst Austrian student-athletes rarely reported any decrease, Italians frequently reported severe decreases in both academic and sports performances.

### Awareness of good practices and possible suggestion for DC implementation (Q48-50)

Only 22% of migrating student-athletes declared to be aware of good practices in DC, mainly from Italy (73%) and Finland (28%). Few of them (*n* = 22) highlighted best practices related to academic flexibility (class attendance, exam sessions, e-learning; 54%), training opportunities (23%), health care (15%), and DC service providers in other countries (15%).

Respondents suggested various DC implementations. These suggestions included increased awareness of DC service providers in student-athletes, increased collaboration between academic institution and sport bodies, clear established DC policies, and financial support and tutorship for organization. At academic level, student-athletes would appreciate an increase in teachers’ awareness of DC so that they could be supported with individualized study programs, availability of online study material, and flexibility in attendance and examinations. At sports level, organizational support for sports facilities and accommodation to allow smooth transition into the new training environment has been suggested. At personal level, student-athletes suggested avoiding setting high goals in the academic field to not interfere negatively with sports performance, establishing a communication with teachers and classmates to keep up with the academic requirements, gaining organizational skills, and always carrying study material.

## Discussion

To develop a European DC culture, efforts have been devoted to integrating sports and educational processes of student-athletes over the last decade [27]. However, whilst the ERASMUS program financially supports the student mobility, no specific DC mobility program exists for student-athletes who face academic and sports challenges during migration in the EU. To suggest support measures for DC migrations, it is necessary to improve knowledge on practices, challenges, supports, and outcomes in DC migrations across countries, especially in consideration of factors that, based on the literature review, can be presumed to influence student-athletes migration and career developments. Because of the different policies and practices in the EU member states, a multi-national approach is needed to reflect the current DC migration situation and to depict the obstacles and requirements for supporting international DC migrations in the EU. For this purpose, it is important to learn from the perspective of the key stakeholder (i.e. student-athlete) because of the known difference between actual and wished supports [28]. For the first time, the current study investigated the mentioned aspects of DC phenomenon of migrating student-athletes via an online survey. The questionnaire was administered to European athletes from five Member States (i.e., Austria, Finland, Germany, Italy, and Slovenia) that have different DC policies in place. Despite the present recruitment procedure not allowing the calculation of the probability and response rates [24], the present data are unique and help to understand the phenomenon of migration within sport and education field from the perspective of student-athletes in multiple EU countries.

The main findings deriving from the descriptive and inferential statistical approaches are highlighted as follows: 1) Migration represents a relevant experience for student-athletes (51% having committed to a migration already), with country-related differences; 2) student-athletes migrate to pursue both academic and sports careers (25%, 32%, and 43% for academic, sports, both careers); 3) female athletes less frequently experience serious decreases in academic performances during migration (females: 27%, males: 31%, decrease expressed by 4 or 5 Likert-points), whereas male athletes perform better in sports (females: 26%, males: 14%, decrease expressed by 4 or 5 Likert-points); 4) financial support is affected by the country of the migrating athletes (Italy: 60%, Finland: 47%, Austria: 47%, Germany: 33%, Slovenia: 81%, reported received financial support); 5) most migrating athletes did not receive tutoring/counselling support (55%), which is in conflict with previously expressed needs [28]; and 6) student-athletes have a limited awareness of DC policies (80%). Based on these findings, some general recommendations can be concluded to enhance DC migration.

At an individual level, migration propensity clusters around the two main education-to-work transitions, which occur at late teens for school-leavers and around 23–25 years of age for university leavers [6]. In DC, migration most frequently occurs around 21–26 years of age, which substantiates that athletes prolong their academic career. In particular, the student-athletes reported difficulties during migration because of the lack of flexibility in the academic field, which is a recommended support in DC [28], as well as the integration into a new sports environment and into a new socio-cultural sphere. Most respondents reported deficits in organizational and/or online support from academic institutions and/or sport bodies. A previous investigation outlined that deficits in organization can cause difficulties in DC in Italy [29]. The combined efforts in DC require efficient organization and management of tasks and requirements. Therefore, proper organization and well-planned, effective schedules can support DC [29]. Another study highlighted the importance of tutoring, e-learning and flexible schedules to improve DC support [28]. Therefore, the implementation of on-site and distance services to facilitate the integration of migrating student-athletes should be prioritized.

The present findings highlight that the migrating student-athletes consider academics and sports equally relevant. In fact, migration can enhance competences and skills or can induce detrimental developments in one of the careers. Whilst DC athletes have the opportunity to enhance their competences and skills of their educational, sports, and cultural development during migration [5], some of them could be overwhelmed by difficulties and experience decreases in academic and/or sports performances. The present findings highlight that less than 30% of respondents experienced relevant decreases in academic performance, with a higher incidence (6%) for male athletes compared with females. Within the globalized culture of elite sport, student-athletes might devote much of their time and energy to sport at the cost of academic achievements. This was corroborated by the low occurrence of decreased sports performance, with a gender-related difference favoring male athletes (13%) compared with females (23%). The gender differences in decreased academic and sports performances may be related to aspects of monetary support and therefore altered commitment to one or the other career path as well as effects on the performance outcomes [12,13]. It is generally known that, in most sports, males receive higher salaries and monetary support compared with females; also the current data showed that more males received financial support and most of this support came from the sports sector. This may result in males focusing more on the sports career and putting more effort into athletic development than females. On the other hand, this may encourage females to commit to the academic career [10] as they experience reduce recognition and reward by the sports sector in comparison with their male peers. To prevent decreases in academic performances, hosting institutions not only should establish harmonized systems with the home institutions and academic study structures with mutual recognizability but are also strongly encouraged to establish specific DC agreements with sports bodies. Compliance of academic and sports requirements is warranted and suggests collaboration between different bodies as a great support [28]. Furthermore, the establishment of specialized tutoring and consulting support is important [28] to prevent migration-related decreases in sports and academic performances. Based on the specific and unique needs of each student-athlete, specialized DC service providers should cooperate with academics and sports, proactively anticipating future requests of assistance and envisioning possible solutions and management strategies [30].

Financial support is a critical issue, which discriminates student-athletes migrating from different Member States. Although the ERASMUS program promotes academic migration, athletes tend to rely more on the sport support, and families showed to help covering costs in many cases. Therefore, a specific ERASMUS DC mobility program should be introduced to financially support migrating student-athletes. In considering that this population tend to relocate for more than 12 months, the time frame of an ERASMUS DC program should be longer than the currently available programs for students. An alternative idea could be the double-funding of student-athletes for their sports and academic careers.

A limitation of this study may be caused by different recruitment strategies applied at the various institutions. This was unavoidable due to the existing country-specific differences in infrastructures and regulations. Whereas some institutions successfully used existing structures to access the target group (e.g. involvement of national authorities and data bases), institutions in other countries where such structures were not available were forced to use more individual approaches and informal ways of communication. To what extent these different strategies may have affected the data is unclear. However, the authors think that the data is thus representative for the countries and the given infrastructures. The recruitment strategies also allowed no calculation of response rates and the size of the overall population in Europe is unclear. Therefore, it remains unclear how the willingness of the voluntary respondents to participate in this study may affected the outcomes (e.g. the proportion that experienced migration already).

Another limitation may be whether the data was representative due to sample size and included countries. First, this investigation obtained data from 5 European countries. These countries represent a suitable selection of different systems and policies in place covering the full spectrum of previously identified DC policies in the EU [20]. Second, the target group was specifically migrating student-athletes at tertiary education level, which is only a portion of all student-athletes. Therefore, the population of this target group is relatively small compared to all student-athletes. As the absolute number of currently existing migrating student-athletes at tertiary level in the EU is unclear, we cannot determine the exact proportion of sample size and population. This should be considered when assessing whether the current sample size (*n* = 245) was statistically representative.

## Conclusions

In consideration of the specific needs and challenges of student-athletes, a DC migration stands out from the general field of migration. The current investigation showed that both chances and challenges in DC migrations cannot be explained by one single factor but by the interaction of multiple factors. This suggests that also solutions cannot focus on a single factor. A wider social context and multiple characteristics of the DC migration need to be considered to implement adequate measures.

To facilitate student-athletes exchanges, a feasible recommendation that would encourage migrations would be to implement better marketing of the academic institutions that offer DC services and support, including helping athletes writing application documents for funding schemes and scholarships. Furthermore, former migrating student-athletes could be engaged to inform the home student-athletes about their experiences and the main factors supporting a DC migration.

Integration strategies and access to locals are recommended because the migration means a change at macro (cultural and social environment), meso (academic and sports environment), and individual level (people with whom the student-athletes work and socialize). In addition, the wide range of challenges and the changing nature of the area points towards the need for better flexibility of programs, clear information, and individual adjustments. Access to technology and technological approaches alone cannot solve the difficulties reported in the current survey and can only support the educational process and other efforts.

In agreement with the ‘EU Guidelines on Dual Careers of Athletes’ [24] (e.g., flexible forms of education, distance learning, and DC services), the partner institutions provided information on the opportunities of flexibility for examinations, individualized study programs, distance learning, and DC services.

Despite the differences in DC policies and services in place at the partner universities, the present findings indicate that a wide range of DC services are feasible, including provided accommodation and sports facilities, DC tutors at academic institutions, professional DC and sports coaches, nutritional advisors, and health care services. The commonalities between universities to support their students’ development could allow for merging and exchanging support structures and services for migrating student-athletes. Infrastructures of e-learning are crucial for migrating student-athletes to keep up with their educational duties. In considering that distance learning programs could require a heavy investment of resources and experience to validate educational material, inter-institutional agreements and clear guidelines for the development and the implementation of high-quality approaches for flexible education are strongly recommended.

## Supporting information

S1 Table Online questionnaire including item number, question, and answer typology. (DOCX)

S1 Data All questionnaire responses from participants including demographic data. (XLSX)
