# Supplementary material for: Multi-national perceptions on challenges, opportunities, and support structures for Dual Career migrations in European student-athletes
Source: PLoS One. 2021 Jun 25;16(6):e0253333. doi: 10.1371/journal.pone.0253333 (PMC8232533; doi:10.1371/journal.pone.0253333)
Supplement: S1 Table — (DOCX) [file pone.0253333.s001.docx]

**S1 Table**

| ITEM | QUESTION | TYPOLOGY |
| --- | --- | --- |
| 1 | What is your gender? | Nominal |
| 2 | What is your age? | Metric |
| 3 | What is your current study level? * | Nominal |
| 4 | What is the total number of training and competition hours per week? * | Metric |
| 5 | What sport do you practice? * | Nominal |
| 6 | What is the country of the club or national championship you are competing for? * | Nominal |
| 7 | What is the national team you are competing for? | Nominal |
| 8 | Are you a student-athletes having moved your residence for sport and/or academic reasons? | Nominal |
| 9 | Did/do/will you receive financial support? * | Nominal # |
| 10 | Did/do/will you receive financial support from the academic field? * | Nominal # |
| 11 | If yes, how helpful was/is it? | Ordinal |
| 12 | Did/do/will you receive financial support from the sport field? * | Nominal # |
| 13 | If yes, how helpful was/is it? | Ordinal |
| 14 | Did/do/will you receive financial support from Dual Career support services? * | Nominal # |
| 15 | If yes, how helpful was/is it? | Ordinal |
| 16 | Did/do/will you receive financial support from other fields? * | Nominal # |
| 17 | If yes, please specify who provided/provides it. | Open |
| 18 | If yes, how helpful was/is it? | Ordinal |
| 19 | How often did/do/will you move your residence for sport and/or academic reasons? | Nominal |
| 20 | For how long did/will you move your residence for sport and/or academic reasons? * | Nominal |
| 21 | Why did/do/will you move your residence? | Nominal+Open |
| 22 | Which city and country are you from? | Open |
| 23 | Which city and country did/will you move to? | Open |
| 24 | Did/do/will you receive tutoring/counselling support? * | Nominal # |
| 25 | Did/do/will you receive tutoring/counselling support from the academic field? * | Nominal # |
| 26 | If yes, how helpful was/is it? | Ordinal |
| 27 | Did/do/will you receive tutoring/counselling support from the sport field? * | Nominal # |
| 28 | If yes, how helpful was/is it? | Ordinal |
| 29 | Did/do/will you receive tutoring/counselling support from Dual Career services? * | Nominal # |
| 30 | If yes, how helpful was/is it? | Ordinal |
| 31 | Did/do/will you receive tutoring/counselling support other fields? * | Nominal # |
| 32 | If yes, how helpful was/is it? | Ordinal |
| 33 | Did/do/will you receive organizational support from the academic field? * | Nominal # |
| 34 | If yes, how helpful was/is it? | Ordinal |
| 35 | Did/do you receive online support from the academic field? * | Nominal # |
| 36 | If yes, how helpful was/is it? | Ordinal |
| 37 | Did/do you receive online support from the sport field? * | Nominal # |
| 38 | If yes, how helpful was/is it? | Ordinal |
| 39 | What difficulties did/do you face caused by your change of residence? | Nominal (multiple) |
| 40 | If “others” were tickled, please specify them. | Open |
| 41 | How severe were/are difficulties you faced at academic level caused by your change of residence? * | Ordinal |
| 42 | How severe were/are difficulties you faced at sports level caused by your change of residence? * | Ordinal |
| 43 | How severe were/are difficulties you faced in other fields caused by your change of residence? * | Ordinal |
| 44 | Did/do you experience decreased performance at academic level caused by your change of residence? * | Ordinal |
| 45 | Did/do you experience decreased performance at sports level caused by your change of residence? * | Ordinal |
| 46 | Please specify any other fields that were negatively affected by your change of residence. * | Open |
| 47 | How severely did/does performance decrease in other fields caused by your change of residence? | Ordinal |
| 48 | Are you aware of good practices to facilitate a change of residence of student-athletes? | Nominal # |
| 49 | If yes, please specify them. | Open |
| 50 | Please provide your suggestions how to facilitate Dual Careers when a change of residence is required? | Open |
